# An Improved Model of Product Classification Feature Extraction and Recognition Based on Intelligent Image Recognition

**DOI:** 10.1155/2022/2926669

**Published:** 2022-08-23

**Authors:** Baiqiang Gan, Chi Zhang

**Affiliations:** ^1^Guangzhou Nanyang Polytechnic College, Conghua 510925, China; ^2^Guangzhou Nanfang College, Conghua 510970, China

## Abstract

With the development of the new generation of technological revolution, the manufacturing industry has entered the era of intelligent manufacturing, and people have higher and higher requirements for the technology, industry, and application of product manufacturing. At present, some factories have introduced intelligent image recognition technology into the production process in order to meet the needs of customers' personalized customization. However, the current image recognition technology has limited capabilities. When faced with many special customized products or complex types of small batch products in the market, it is still impossible to perfectly analyze the product requirements and put them into production. Therefore, this paper conducts in-depth research on the improved model of product classification feature extraction and recognition based on intelligent image recognition: 3D modeling of the target product is carried out, and various data of the model are analyzed and recorded to facilitate subsequent work. Use the tools and the established 3D model tosimulate the parameters of the product in the real scene, and record them. Atthe same time, various methods such as image detection and edge analysis areused to maximize the accuracy of the obtained parameters, and variousalgorithms are used for cross-validation to obtain the correct rate of the obtaineddata, and the standard is 90% and above. Build a data platform, compare simulated data with display data by software and algorithm, and check by cloud computing force, so that the model data can be as close to the parameters of the real product as possible. Experimental results show that the algorithm has high accuracy and can meet the requirements of different classification prospects in actual production.

## 1. Introduction

Physical manufacturing has always been the foundation of national economies, so the development of indigenous manufacturing is a top priority for both developed and developing countries.

In recent years, with the continuous development of artificial intelligence technology, the manufacturing industry is also constantly introducing new technologies. The first is the regions represented by developed countries such as Europe and North America. Countries in these regions have vigorously promoted the strategy of returning manufacturing to their own countries in recent years, trying to resist the wave of economic globalization while applying emerging technologies to their own manufacturing industries. At the same time, it can limit the outflow of advanced technology to the greatest extent, so as to improve the competitiveness of its own manufacturing products on a global scale, and try to monopolize technology. Previously, the spillover of manufacturing industry in Europe and the USA was mainly due to the consideration of labor cost. However, the arrival of new manufacturing forms is likely to greatly weaken the position of low-grade cheap labor in manufacturing industry, thus making some labor-oriented countries lose their only advantages in the wave of new industrialization.

After more than 40 years of reform and opening up, China's industrial level has been greatly improved, and China has successfully become one of the world's largest manufacturing countries [1]. However, the new wave of industrialization still has a great impact on China's manufacturing industry. As we all know, due to the implementation of China's reform and opening up strategy in the 1970s and 1980s, it is since then that a considerable number of manufacturing factories have been introduced in China, but China's advantage has always been a large number of cheap labor market, and the competitiveness in technology is particularly weak. Therefore, although the scale of China's manufacturing industry is in the forefront of the world, the technology of manufacturing industry is always not worthy of today's manufacturing scale. Another problem that cannot be ignored is that most of the foreign-funded manufacturing industries transferred to China are domestic low-end manufacturing industries, which is not conducive to China's technological accumulation. Therefore, China's manufacturing industry is facing the dual challenges of industrial transformation and industrial upgrading. Also to put forward the corresponding strategy in our country, in the information technology in manufacturing practice closer now, again in manufacturing upgrading of key nodes, our country needs to catch up with the pace of the developed countries, break the technical barriers of developed countries, complete its manufacturing technology upgrading, and thus in manufacturing occupy the position of the world.At present, manufacturers who actively follow the trend of the times pay more and more attention to the realization of intelligent manufacturing, and put it into the real production line. The diversity of products makes the classification and identification of products more difficult. In response to this problem, the method proposed in this paper is compared with the analysis method using cloud data to improve the accuracy of recognition between the model and the actual product. Users draw the STL model of the product on the cloud according to their own needs and upload the product data to the manufacturer for production [2]. Industrial cameras are used to collect images of the products and match them with cloud data so as to identify and classify the products. Therefore, in order to solve these problems, this paper makes an in-depth study on the improved model of product classification feature extraction and recognition based on intelligent image recognition.

In addition, a method of image preprocessing is proposed. Finally, the paradigm of contour extraction is determined and the method of contour feature description is studied. Through comparison and analysis, the contour feature description with high recognition accuracy was selected.

## 2. The Research Background

### 2.1. Vision and Machine Vision

Vision usually undertakes very important tasks of detection and inspection in the manufacturing industry. Since vision can obtain a large amount of information, vision is often used as a reliable tool in the noninformation age [3]. With the development of manufacturing industry, the accuracy and precision requirements of manufacturing industry are getting higher and higher. The accuracy of information provided by ordinary human vision does not meet the requirements of today's products, while the labor cost is increasing; therefore, machine vision is proposed by scientists and gradually applied in practice. Machine vision recognition was born in the 1950s, and it first uses the camera to record the current state of the machine and then analyzes the speed and direction of the machine's movement based on the captured images. It first used a camera to record the current state of the machine, then analyzed the speed and direction of the machine motion based on the captured images, and then regulated the next machine motion [4]. In the 1960s, the study of machine vision recognition evolved from two-dimensional to three-dimensional. First, American professors believed that, just as polygons can be understood by breaking them into triangles of different sizes, if images of physical objects are taken and processed by a computer program and broken down into more easily understood and analyzed basic three-dimensional structures such as cubes, spheres, cylinders, and vertebrae, and their coordinates and dimensions are described in mathematical language, and if simple regular three-dimensional structures can be extended to more complex three-dimensional structures, machine stereo vision recognition will have a wider application [5]. The above is the theoretical basis for the gradual introduction of machine vision into manufacturing work.

### 2.2. Research Status

After the 1960s, the research on the three-dimensional effect of machine vision provided objective conditions for future machine recognition and learning of three-dimensional objects in the objective world and also made the application of machine vision possible [6]. Since then, a large number of scientists have joined the research on machine vision, and the related theories have progressed by leaps and bounds. However, since most of the analysis work needs to be done by computers, the upper limit of computer performance determines the upper limit of theoretical analysis of machine vision, and if the current cognitive limit is to be broken, the computing power of computers must be improved first. Until the 1990s, due to the breakthroughs in mathematics, new algorithmic logics were proposed and established that allowed computers to surpass their previous computational power, which meant that computer computing power could finally support the gradual application of machine vision to practice [7].

However, China's machine vision research was influenced by China's national conditions, and the research in this area started late and developed slowly until the 1980s, which was the period when China opened up to the outside world and widely absorbed foreign cultures. In the 21st century, China's machine vision benefited from various “Internet+” policies to develop rapidly [7, 8]. In recent years, with the development of artificial intelligence and the programmatic documents such as “intelligent manufacturing pilot demonstration action implementation plan,” the manufacturing industry has been promoted to the direction of intelligence, and the domestic research on machine vision technology has also continued to achieve new results. Although the scale of the domestic intelligent manufacturing industry is developing rapidly, in the actual application of the production line, the scale is small, the degree of automation is low, the effect needs to be improved, and there is still a certain distance abroad, really in the application, such as workpiece surface quality inspection, electronic device manufacturing, and other fields which still do not completely use intelligent inspection system. In addition, there are many other challenges: a large number of manufacturers have begun to seek new ways to reduce costs and improve efficiency, and this new way is to use machine recognition technology to replace manual quality inspection and product classification [9].

The need to reduce production costs and increase productivity, as well as the expectations of people, has driven the popularity of machine vision technology in practical applications [10]. Nowadays, more and more research institutions, universities, etc., are focusing on machine vision research, investing a lot of time, money, and effort in research, and have made great progress in applications in industry, agriculture, and medicine, which are expected to catch up with foreign research [11]. The first is the application on various electronic components; at present, machine vision is mainly used to check whether the PCB printing is complete, whether the holes are missing, etc. This kind of problem, which is difficult to check precisely under human vision, happens to be the part where machine vision is good at. In addition, our scientists have also used machine vision to measure the density of industrial-grade finished products, etc., and have achieved some results. In agriculture, machine vision is also used to examine the characteristics of plants and analyze their color to screen for quality with a high degree of accuracy in the end. However, at present, the scope of most applications of machine vision in China is still limited to the inspection of appearance, whether the appearance of qualified, uneven, or stained, ultimately based on a simple analysis of the appearance of three-dimensional data, and in a comprehensive view, there are still shortcomings compared with the world's advanced machine vision application technology.

## 3. Materials and Methods

**Figure 1:**
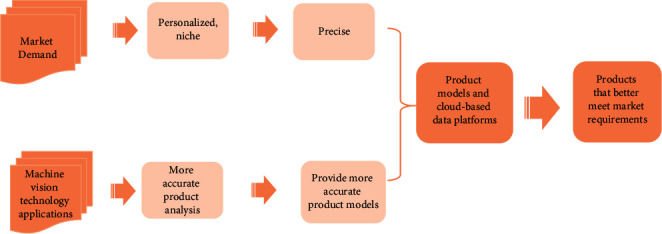
Market demand and technology application implementation framework.

From the supply side, the transformation and upgrading of this industry of manufacturing have become concerns in various fields at home and abroad in recent years, and at the same time, the current technology can be put into application because of the rapid development of information technology, which also provides the technical basis for the update of manufacturing technology.From the demand side, the current demand for products is increasing, and the manufacturing technology used in the past for a large number of wholesales has been unable to keep up with the development trend of the current era. In order to meet market demand, it is also necessary to match machine vision data with customer demand data to more accurately meet customer needs. Therefore, this paper designs a market demand and technology application implementation framework based on image recognition technology for the common requirements of the supply side and the demand side to meet the supply side and the demand side requirement situation as shown in Figure 1.

The steps on how to compare and analyze the product model with the cloud data for subsequent more detailed processing are as follows: first, preprocessing is performed to reduce the impact caused by the environment, and then contour features are extracted and described. At the same time, the STL model uploaded from the cloud is parsed and the extracted boundaries are extracted for contour features. Then, the similarity between the product image and the contour of the cloud-based STL model is compared and matched. Finally, the results of classification and recognition are output.

### 3.1. STL-Based Product Matching Template Generation

It mainly refers to parsing the data of STL models uploaded by users. The current method of identifying product types in the actual production line is mainly using identification templates for product matching, and because the process of identification templates is very complicated and the number of templates is limited, this method can only identify a small number of product types in the production process of the same production line, which is difficult to meet user requirements [12]. Therefore, in order to solve the problem of difficulty in manufacturing product matching templates, this section proposes a method to generate matching templates directly using 3D models of products in the cloud. Companies and individuals set the template generation perspective in OpenGL, build a three-dimensional model of the product according to the desired functionality, finally save it as an STL-type file, and then submit the order to the manufacturer for mass production of the product. After receiving the 3D model uploaded by the user, the topological information of the product needs to be completely read, and the read data need to be parsed and processed [13].

### 3.2. Image Preprocessing and Contour Extraction

Before product classification and recognition, the product images should be preprocessed because in the process of image acquisition, they may be influenced and interfered by the external environment, such as the brightness of the light and the stability of the camera. These interferences may lead to large errors between the acquired images and the actual products, which will inevitably result in overfitting or low accuracy when they are transferred to the system. These disturbances may lead to large errors between the acquired images and the actual products, which may result in overfitting or low accuracy when transmitted to the system, and subsequent image segmentation, recognition, and classification may produce deviations and affect the results of image analysis and processing [14]. In order to solve such problems, the acquired images must be preprocessed with noise removal and enhancement. The contour extraction mainly utilizes the Canny edge detection operator. When performing Canny edge detection, since the input product image is an RGB color image, the range of values for each pixel is too large leading to a very complex calculation, which can be simplified by using a binarized image form in digital image processing, and the input RGB color image is first Gaussian smoothed to convert the color image into a grayscale image [15]. Then, the gradient of the pixels in the grayscale image is calculated; if the gradient between adjacent pixels is zero, these two pixels belong to the same region; if the gradient is not zero, these two pixels belong to different regions. However, the boundary of the region formed in this way is a stepped dash, which needs to be processed in some way using a fitting algorithm, and then two different critical values are used to connect the dividing lines of neighboring pixels; thus, a smooth curve is obtained and the detection results of the grayscale image are finally output and displayed [16]. Meanwhile, the Canny algorithm can be optimized in multiple stages and has good performance in detecting the edges of image pixel regions. After the edge detection of the image using the Canny edge detection operator, the contour extraction of the image is required. In this paper, we choose the contour tracking algorithm to extract the contour features of the image. The algorithm first searches for a point on the image edge as the starting position, and then finds other contour points based on the gradient value of the pixel, tracks the edge, and obtains the complete contour of the image. The gradient of the pixels inside the same contour is the same after the contour tracking is finished [17].

**Figure 2:**
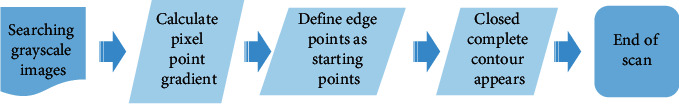
Contour tracking algorithm flow.

The contour tracking algorithm has the following main steps: first, the preprocessed grayscale image is carpet searched, the gradient of the current pixel point is calculated, it is judged whether the pixel point is a point on the edge, and if this point is judged not to be a point on the edge, the search continues until the point on the edge is searched, which is defined as the starting point and given a tracking label; after the starting point is found, the contour line strip, due to its continuity, it is only necessary to search in the adjacent points of that point. Therefore, the starting point and scanning direction are redefined, the searched contour points are the new starting points, the points are searched one by one in the clockwise direction, and the above judgment method is used to determine whether these points are contour points or not. If there is a point with the same coordinates as the initial point, the search is finished; i.e., all points form a closed and complete contour; otherwise, the search continues according to the above steps until a closed and complete contour is formed. The specific flow is shown in Figure 2.

Although the above algorithm is efficient and simple, in order to make the contour tracking and extraction of the image more effective, the Find Contours function is used in the OpenCV computer vision development library for contour extraction.

### 3.3. Contour Feature Description

**Figure 3:**
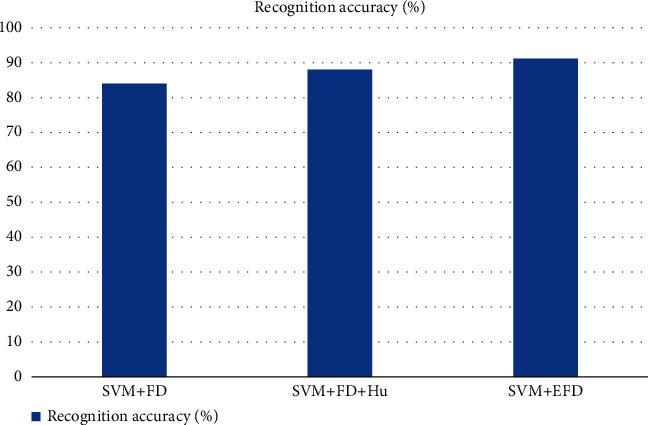
Recognition accuracy of Fourier transform algorithms.

To achieve product-specific recognition and classification, contour features need to be described in a way that is influenced by parameters such as the initial point, size, and orientation of the contour, so Fourier features must be normalized. In this paper, Fourier feature description methods are investigated and the recognition accuracy of SVM-based Fourier transform algorithm, Hu-based Fourier feature description algorithm, and SVM-based elliptic Fourier transform algorithm is compared [18]. The comparison of recognition accuracy data of several types of algorithms is shown in Figure 3.

From Figure 3, it can be seen that the SVM-based elliptic Fourier transform algorithm has the highest recognition accuracy than the SVW-based Fourier transform algorithm and the Hu-based Fourier transform algorithm, so this paper will use the SVM-based elliptic Fourier transform algorithm for contour feature extraction and description to obtain a certain product classification accuracy.

## 4. Results and Discussion

In this section, experiments are conducted and results are obtained based on the product contour extraction and template matching methods proposed in the previous section. Firstly, the cloud-based STL model uploaded by users is parsed for data, and the contour features are identified and extracted to generate the matching templates for products. Then, the machine vision inspection platform is built, the camera is calibrated according to the accuracy requirements and the size of the product to be inspected, a suitable chip and lens are selected for the camera, and image acquisition is performed. Finally, contour extraction and feature description are performed on the acquired products, and the acquired images are matched with the cloud-based STL model to complete the final product identification and classification.

### 4.1. Selection of Industrial Cameras

The first step of product identification is to acquire images, and it can be said that the camera is the most important instrument in the whole process. Different imaging modes can be formed by matching the camera with the lighting method [19]. The simplest is the reflection mode, which is illuminated by a point light source, and the camera takes pictures directly to the target area, but reflections occur when the target object has a shiny surface. The diffuse mode uses a surface light source, thus providing more uniform illumination and reducing reflections. Transmission method is to place the object between the light source and the camera, the use of shadow imaging. The scattering method uses a shade to scatter the light source. In this experiment, a diffuse reflection approach was used to uniformly illuminate the target object with a white surface light source [20]. Then, the camera uses the lens to focus the light to produce an image. In order to ensure the consistency of the captured image and a certain detection accuracy, there are certain requirements for the parameters of the camera. Among them, the parameters that have the greatest impact on the specific imaging effect are the sensor, the resolution, and the lens of the camera, respectively. This section will analyze specifically from the above three aspects. First is the need to choose the appropriate digital imaging chip as the camera's light sensor. According to the working principle, digital imaging chips can be divided into two types: CMOS and CCD. CMOS cameras have complex transmission paths and are therefore disturbed by more noise, resulting in easy distortion of data and lower accuracy; the CCD camera is very similar to the memory circuit in transmitting data, and the images taken during the transmission process are almost distortion-free and highly accurate. Therefore, the CMOS camera is used in this experiment for subsequent image acquisition. In addition, the resolution of the camera is also an important parameter, which is related to the accuracy of product identification. The higher the resolution of the camera, the higher the accuracy of the product recognition. However, considering the cost, the resolution should not be chosen in pursuit of the detection accuracy, but the actual size of the product to be detected should be considered, and it is enough to accurately record the contour characteristics of the product. It is also necessary to choose the frame rate of the camera and the form of camera scanning imaging. In this experiment, the size of the products to be inspected does not exceed 12 cm, so to ensure that the products to be inspected can be completely photographed, and the camera's photo range is set.

In this experiment, the size of the products to be tested does not exceed 12 cm. The last thing you need to choose is the lens of the camera. The focal length of the lens and other parameters will have an impact on the imaging effect and quality, and the main factors to consider when choosing a lens are contrast, depth of field, and resolution. Therefore, the importance of lens selection is no less than the choice of digital imaging chip. In this experiment, considering the shooting distance and the target object, a fixed-focus lens is selected, whose model is JMSF0618-5A with a focal length of 8 mm. After the camera was selected, a light weight 2036A aluminum plate was used to build the bottom support of the camera on the production line and connect the camera to the computational processing system.

**Figure 4:**
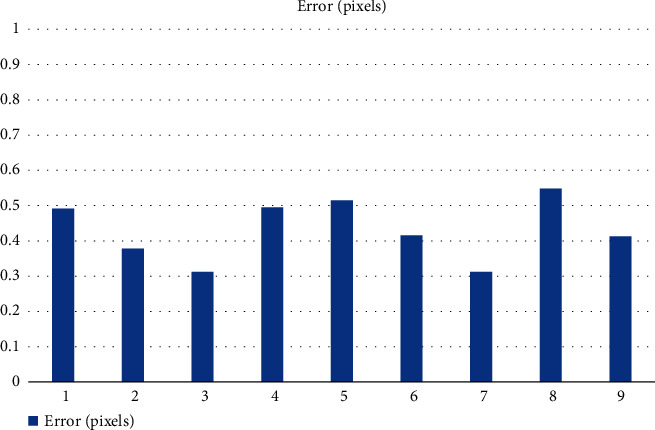
Calibration error.

Finally, the internal and external reference matrices of the camera were extracted, then the target object was photographed with the camera, and the parameters of the camera were modified and optimized according to the actual photographs taken. First, the calibration images are taken. The number of calibration images determines the accuracy of camera calibration, and Zhang Zhengyou's calibration method usually requires no less than three images. However, the number of calibration images should not be too many; otherwise, it will affect the calibration efficiency. In order to achieve higher calibration accuracy and ensure the calibration efficiency, the calibration algorithm is written using the OpenCV database and Visual Studio 2016 software, and nine images with different shooting angles are used for calibration. Then, the coordinates of each pixel on the calibrated image need to be calculated, the color of that point is also represented and extracted numerically, and the color values and coordinates are corresponded one by one to generate a two-dimensional matrix. A two-dimensional matrix is likewise generated from the actual photographs taken. A program is written to compare the values of the corresponding positions of the two matrices one by one, and then the parameters of the industrial camera are normatively corrected to determine the final calibration results. The calibration error is shown in Figure 4.

**Figure 5:**
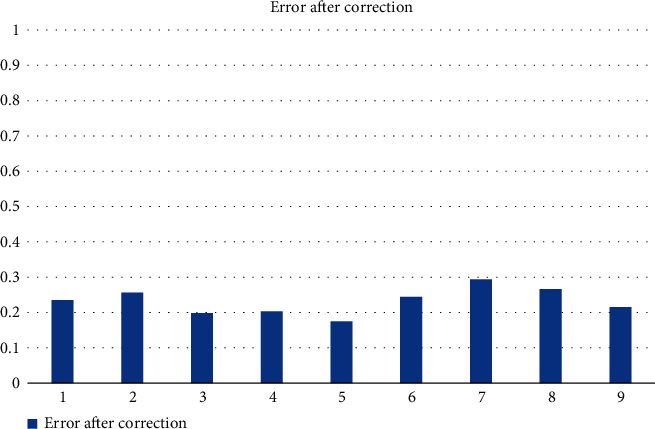
Corrected calibration error.

From the calibration errors shown in Figure 4, it can be seen that the calibration errors of the nine calibration images are too large, all of them exceed 30%, and the largest one even reaches 50%. This will lead to excessive distortion and seriously affect the accuracy of product detection, so the calibration camera must be corrected so as to reduce the pixel error. The calibration results were used to correct the selected 9 images, and the corrected errors are shown in Figure 5.

From Figure 5, it can be seen that the calibration error is significantly reduced after the correction of the calibration results, and the calibration error of the nine calibration images is controlled below 30%, which almost does not affect the recognition and classification work of the system. This shows that it is very necessary to correct the calibration results.

### 4.2. Product Classification Experiment

This paper proposes an experimental validation of the proposed feature extraction and description algorithm on a real production line, and the specific product identification and classification process is shown below.

Uploading STL models of products in the cloud in order to ensure the generality of the experimental results, this experiment uploads five STL models with different structures in the cloud. Then, the data analysis and processing of the STL models in the cloud are performed to generate.

The data from the cloud STL models are then analyzed and processed to generate the matching templates for detection. At the same time, the data of the models were uploaded to the 3D printer to obtain the GCODE files of the models for product production. The parameters of the 3D printer, such as the specific information of the 3D printer, need to be set before 3D printing. Then, we set up a machine vision product recognition and classification platform, set up a camera at a suitable location on the production line, and use the camera to upload images of products to the system for contour extraction and feature description. After getting the matching template of the product, we use Visual Studio 2016 software to write generate descriptor of contour project to extract the features of the matching template, and save the feature data generated by the project in the format of a text document. At the same time, the contours obtained from the product images are also extracted and saved in text file format. Finally, the SVM-based elliptic Fourier algorithm is used to identify and classify the products.

**Figure 6:**
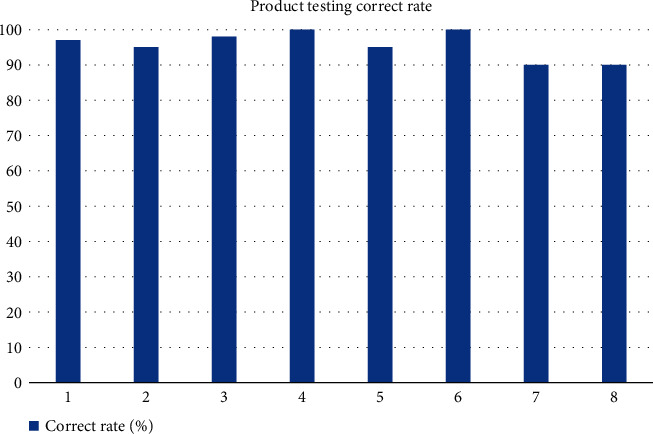
Product detection accuracy rate.

The SVM-based elliptic Fourier algorithm is trained to improve the accuracy of the final product recognition and classification. After obtaining the actual images and matching templates, the MATLAB 2020a software is used to extract features from the sample library and classify the data with similar features into one class. After several training sessions, the parameters of this algorithm will be optimized and the resulting model will be more complete, allowing for more accurate product recognition and classification on the production line. The product recognition and classification system is built on the production line to sort the products. The recognition platform is built on the main conveyor belt, a small section of selected industrial camera is installed above the conveyor belt, and LED backlight with certain light intensity is installed at a suitable location near the camera to fill the light for the products. The product images are sent to the system for feature extraction and description, matching with the target type, and then the product type is detected and sent to the corresponding sub-track. The results of the product detection are shown in Figure 6.

**Figure 7:**
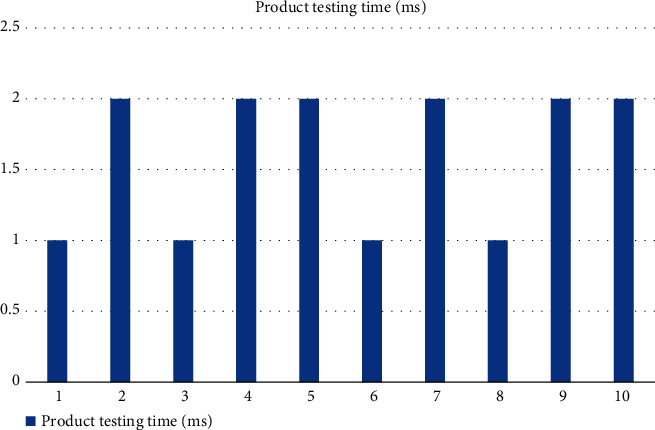
Product detection time.

From Figure 6, it can be seen that the accuracy rate of product classification and identification reaches more than 90% each time, and the experimental results fully verify the effectiveness of the proposed algorithm, which can accurately identify and classify products. The product detection time of the eight experiments is shown in Figure 7.

From Figure 7, we can see that the product detection time of each experiment is very short, no more than 2 ms, and the average product classification time is only 1.5 ms. This shows that the proposed algorithm can quickly classify and identify products, which ensures a certain efficiency and can be put into practical application. Therefore, the sequence of this experiment is to collect the required digital chips, lenses, and other hardware, and build a machine vision product recognition and classification platform. Finally, the contour extraction and feature description algorithms were verified, and the product recognition and classification were accurate enough to achieve small-scale production.

## 5. Conclusion

With the development of artificial intelligence, the manufacturing industry has also entered the era of intelligent manufacturing, and people have higher and higher requirements on the technology, industry, and application of product manufacturing. At present, some factories have introduced intelligent image recognition technology into the production process in order to adapt to the needs of customers' personalization, but the current algorithms have low adaptability to product types. Therefore, to address these problems, this paper conducts an in-depth study on the product classification feature extraction and recognition improvement model based on intelligent image recognition. The first is to propose a more convenient and quicker way to generate matching models, that is, to use the STL models provided by customers for analysis in the cloud: users upload STL models in the cloud according to their needs, and by completely reading the topological information of the 3D graphics such as points, lines, and surfaces of the STL models in the cloud, parsing and processing the read data, and finally generating matching templates. This method of generating matching templates is more concise compared to the traditional method of creating recognition templates. Then, image preprocessing is performed.In the process of image acquisition, it may be affected and interfered by the external environment, such as the brightness of the light, the stability of the camera, etc. These interferences may cause large errors between the acquired image and the actual product. And there are unnecessary noises and other interferences in the collected images, which may cause deviations in subsequent image segmentation, recognition and classification. In order to reduce the error, image preprocessing is required. In this paper, image filtering methods and edge detection algorithms are investigated, and the final decision is made to use the Canny edge detection operator for contour extraction. In addition, the contour feature description method is studied. The current commonly used Fourier transform algorithms are compared and analyzed. Finally, the algorithm with high recognition accuracy is selected for contour characterization of the product.

## Data Availability

The labeled dataset used to support the findings of this study is available from the corresponding author upon request.
